# COVID-19 patient serum less potently inhibits ACE2-RBD binding for various SARS-CoV-2 RBD mutants

**DOI:** 10.1038/s41598-022-10987-2

**Published:** 2022-05-03

**Authors:** Daniel Junker, Alex Dulovic, Matthias Becker, Teresa R. Wagner, Philipp D. Kaiser, Bjoern Traenkle, Katharina Kienzle, Stefanie Bunk, Carlotta Struemper, Helene Haeberle, Kristina Schmauder, Natalia Ruetalo, Nisar Malek, Karina Althaus, Michael Koeppen, Ulrich Rothbauer, Juliane S. Walz, Michael Schindler, Michael Bitzer, Siri Göpel, Nicole Schneiderhan-Marra

**Affiliations:** 1 NMI Natural and Medical Sciences Institute at the University of Tübingen, Markwiesenstrasse 55, 72770 Reutlingen, Germany; 2 Pharmaceutical Biotechnology, Eberhard Karls University, Tübingen, Germany; 3 Department Internal Medicine I, University Hospital Tübingen, Otfried-Müller-Strasse 10, 72076 Tübingen, Germany; 4 Department of Anesthesiology and Intensive Care Medicine, University Hospital Tübingen, Tübingen, Germany; 5 Institute for Medical Microbiology and Hygiene, University Hospital Tübingen, Tübingen, Germany; 6 German Center for Infection Research (DZIF), Partner Site Tübingen, Tübingen, Germany; 7 Institute for Medical Virology and Epidemiology, University Hospital Tübingen, Tübingen, Germany; 8 Center for Personalized Medicine, Eberhard Karls University, Tübingen, Germany; 9 Institute for Clinical and Experimental Transfusion Medicine, University Hospital Tübingen, Tübingen, Germany; 10 Department of Internal Medicine, Clinical Collaboration Unit Translational Immunology, German Cancer Consortium (DKTK), University Hospital Tübingen, Tübingen, Germany; 11 Department of Immunology, Institute for Cell Biology, University of Tübingen, Tübingen, Germany; 12 Cluster of Excellence iFIT (EXC2180) “Image-Guided and Functionally Instructed Tumor Therapies”, University of Tübingen, Tübingen, Germany; 13 Dr. Margarete Fischer-Bosch-Institute for Clinical Pharmacology, Robert Bosch Center for Tumor Diseases (RBCT), Stuttgart, Germany

**Keywords:** Immunochemistry, Immunology, Adaptive immunity, Immune evasion, Infectious diseases, Vaccines

## Abstract

As global vaccination campaigns against SARS-CoV-2 proceed, there is particular interest in the longevity of immune protection, especially with regard to increasingly infectious virus variants. Neutralizing antibodies (Nabs) targeting the receptor binding domain (RBD) of SARS-CoV-2 are promising correlates of protective immunity and have been successfully used for prevention and therapy. As SARS-CoV-2 variants of concern (VOCs) are known to affect binding to the ACE2 receptor and by extension neutralizing activity, we developed a bead-based multiplex ACE2-RBD inhibition assay (RBDCoV-ACE2) as a highly scalable, time-, cost-, and material-saving alternative to infectious live-virus neutralization tests. By mimicking the interaction between ACE2 and the RBD, this serological multiplex assay allows the simultaneous analysis of ACE2 binding inhibition to the RBDs of all SARS-CoV-2 VOCs and variants of interest (VOIs) in a single well. Following validation against a classical virus neutralization test and comparison of performance against a commercially available assay, we analyzed 266 serum samples from 168 COVID-19 patients of varying severity. ACE2 binding inhibition was reduced for ten out of eleven variants examined compared to wild-type, especially for those displaying the E484K mutation such as VOCs beta and gamma. ACE2 binding inhibition, while highly individualistic, positively correlated with IgG levels. ACE2 binding inhibition also correlated with disease severity up to WHO grade 7, after which it reduced.

## Introduction

Neutralizing antibodies (Nabs) prevent infection of the cell with pathogens or foreign particles by neutralizing them, eliminating a potential threat and rendering the pathogen or particle harmless^1^. The longevity of a Nab response has important implications for immune protection and vaccination strategies. In SARS-CoV-2, Nabs interfere with the cell entry mechanism primarily by blocking the interaction of the receptor binding domain (RBD) with the human cell receptor angiotensin converting enzyme 2 (ACE2)^2,3^. The RBD of SARS-CoV-2 is target of approximately 90% of the neutralizing activity present in immune sera^4^, with a lack of Nabs correlating with risk of fatal outcome^5,6^. Passive transfer of Nabs through convalescent serum or as monoclonal antibodies have been shown to provide protection from infection^7–9^, with several Nabs drugs granted emergency use authorization by the U.S. Food and Drug Administration^10–13^.

Since the first documented infections in Wuhan China^14^, SARS-CoV-2 has continually evolved, with the emergence of global variants of concern (VOCs) being of particular importance. As of this moment, the WHO lists the alpha (B.1.1.7)^15^, beta (B.1.351)^16^, gamma (P.1)^17^, delta (B.1.617.2)^18^ and omicron (B.1.1.529)^19^ strains as VOCs^20^, in addition to further variants of interest (VOIs) such as lambda (C.37) ^21^. The emergence and disappearance of variants and continual mutation of SARS-CoV-2 is of particular relevance for vaccine development, as all currently licensed vaccines^22–25^ only elicit an immune response against the spike protein based on the original Wuhan-Hu-1 isolate (hereon referred to as “wild-type”)^26,27^. Several studies have already found that both convalescent and post-vaccinated sera have lower neutralization capacities against beta and gamma VOCs^28–30^. Of particular concern are mutations on amino acid residue (aa) 484 (e.g. E484K), which seem to confer escape from vaccine control, with an additional mutation on aa 501 (e.g. N501Y) increasing this effect^31^.

In order to lead development of new vaccines and safely lift social restrictions, definitive correlates of protective immunity are necessary^32^. The gold standard for Nabs assessment are virus neutralization tests (VNTs), however these require live infectious virions which must be handled in biosafety level 3 (BSL3) laboratories, as well as access to variant strains of SARS-CoV-2. In this study, we developed and applied RBDCoV-ACE2, a multiplex ACE2-RBD inhibition assay based upon the antibody-mediated inhibition of ACE2-RBD binding. This automatable assay enables simultaneous screening of serum samples for the presence of Nabs against a great number of VOCs/VOIs in a single well, making it a time-, material- and cost-effective alternative to live VNTs or classical ELISAs. Following in-depth validation of the assay, we analyzed the IgG antibody response and ACE2 binding inhibition of 266 serum samples from 168 COVID-19 patients with mild to severe disease progression towards eleven different SARS-CoV-2 variant RBDs including the alpha, beta, gamma and delta VOCs.

## Results

### ACE2-RBD inhibition assay (RBDCoV-ACE2) validation

To investigate the inhibition of ACE2 binding by SARS-CoV-2 VOCs, we developed and established a high-throughput bead-based multiplex ACE2-RBD inhibition assay (from here on referred to as “RBDCoV-ACE2”). This assay mimics the ACE2-RBD interaction and thereby detects the presence of Nabs against SARS-CoV-2 that inhibit this interaction. At the time of experimentation, RBDCoV-ACE2 contained the RBDs of SARS-CoV-2 wild-type and 11 different variants (alpha, beta, gamma, epsilon, eta, theta, kappa, delta, lambda, Cluster 5 and A.23.1).

**Figure 1:**
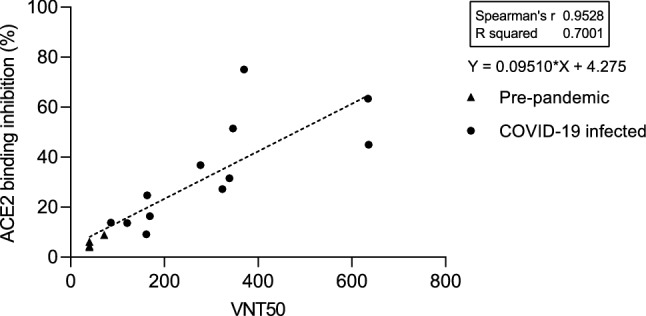
Comparison between RBDCoV-ACE2 and a virus neutralization test (VNT). Serum samples (n = 16) of pre-pandemic (n = 4) and COVID-19 convalescent (n = 12) individuals were measured using both assays and analyzed by linear regression. The equation of the dashed regression line is shown next to the graph. VNT results are depicted as half-maximal inhibiting serum dilutions (VNT50), RBDCoV-ACE2 results are shown in percentage inhibition of ACE2 binding. Correlation analysis was performed after Spearman and the correlation coefficient r is shown.

To validate the assay, we both compared performance to a standard VNT (Fig. 1), as well as completed technical validation to FDA bioanalytical guidelines verifying reagent stability, assay precision, freeze–thaw stability and parallelism (Figure S1). An assay validation sample set of 16 samples (12 convalescent, 4 pre-pandemic) was measured by VNT against wild-type and with RBDCoV-ACE2. The results of both assays showed a strong correlation (Spearman’s rank 0.95), confirming that RBDCoV-ACE2 is measuring neutralizing antibodies specifically (Fig. 1). Technical validation performed with a set of 6 samples (4 vaccinated, 1 infected, 1 pre-pandemic) confirmed that RBDCoV-ACE2 is highly reproducible, as seen by the low intra- and inter-assay variation (all CVs under 5% and 7% respectively, Figure S1a and b, Table S3). ACE2 buffer was shown to be stable both in storage (4 °C) and at room temperature (21 °C), with minimal loss in performance compared to freshly prepared buffer (Figure S1c). Similarly, the biotinylated ACE2 stock solution showed high freeze–thaw stability (all CVs under 13%, Figure S1d). Parallelism was used to optimize the assay conditions and to ensure that the ACE2 concentration was in linear range (Figure S1e). Percentage coefficients of variation (%CV) of all technical validation experiments for every analyzed sample are summarized in Table S3. Lastly, to ensure that the multiplex nature of the assay was not causing competition between beads for ACE2 which would have resulted in artificially deflated values, the assay was performed as both a singleplex (for all VOCs) and multiplex with 24 samples (19 COVID-19 infected, 5 pre-pandemic), with no difference in performance between the two bead compositions found (Figure S2).

### RBDCoV-ACE2 comparison to commercially available assay

**Figure 2:**
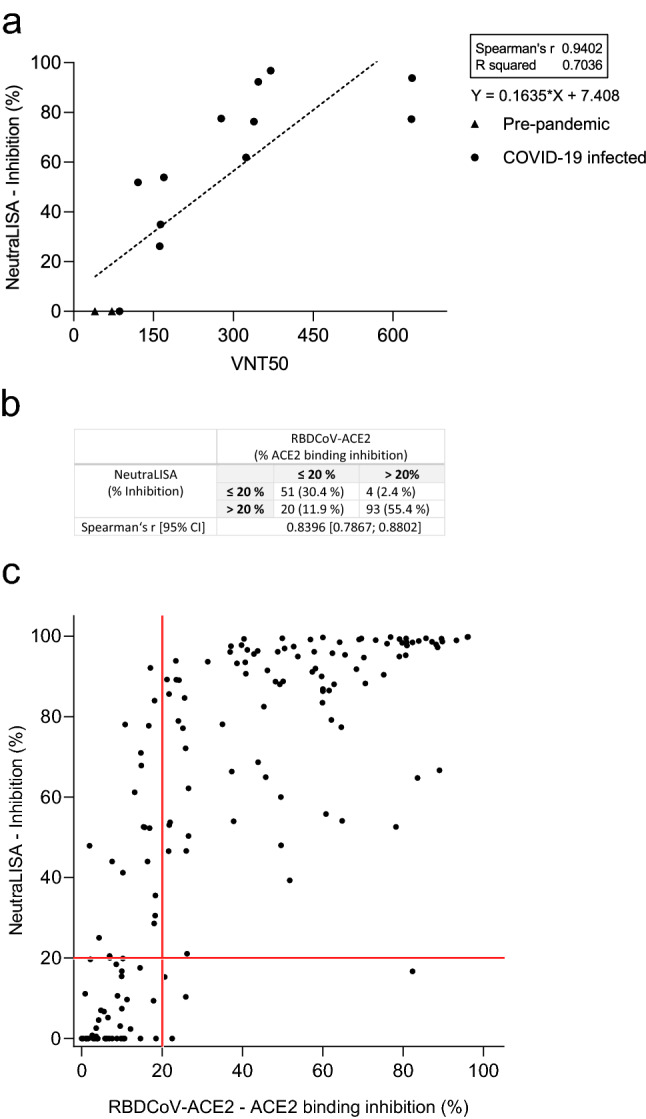
Correlation between SARS-CoV-2 NeutraLISA and VNT and comparison to RBDCoV-ACE2. (**a**) Correlation and linear regression between NeutraLISA and VNT results for pre-pandemic (n = 4) and COVID-19 infected (n = 12) samples. Correlation analyses were performed after Spearman and correlation coefficients r are shown. (**b**) Descriptive statistics of the (**c**) correlation between NeutraLISA and RBDCoV-ACE2. One sample from each individual (n = 168) was measured using both assays. Correlation was calculated after Spearman. Samples were classified as being negative (non-neutralizing) if they had a value below 20% (red lines).

To compare RBDCoV-ACE2 performance to a similar commercially available inhibition assay, we initially tested our assay validation sample set on NeutraLISA and compared its performance to the VNT (Fig. 2a). While the results of the two assays did correlate (Spearman’s rank 0.94), the NeutraLISA appeared to reach a plateau and saturate, as seen by the high inhibition percentage for all samples with a VNT50 greater than 350. To confirm this plateau effect, we analyzed a subset of samples from our COVID-19 sample collection on both RBDCoV-ACE2 and the NeutraLISA, finding that while a strong correlation between the results existed (Spearman’s rank 0.84, Fig. 2b), the saturation plateau was still present (Fig. 2c). This suggests that RBDCoV-ACE2 has a more dynamic range and better resolution, especially in the higher inhibition percentages. When classifying samples as being either positive or negative, samples with an inhibition percentage under 20% are considered negative for the NeutraLISA^33^. As both assays detect bound ACE2, we implemented a similar cut-off for RBDCoV-ACE2. Overall, 30.4% of samples (51/168) were considered negative in both assays, while a further 55.4% (93/168) were considered positive in both (Fig. 2b). Of the remaining samples, 4 (2.4%) exceeded 20% binding inhibition only in RBDCoV-ACE2, while 20 (11.9%) exceeded 20% inhibition in the NeutraLISA only. Overall, the stronger correlation between RBDCoV-ACE2 and VNT (Fig. 1) compared to NeutraLISA and VNT, as well as the increased dynamic range, proves RBDCoV-ACE2 has superior assay performance.

### ACE2 binding inhibition is reduced for mutant RBDs

**Figure 3:**
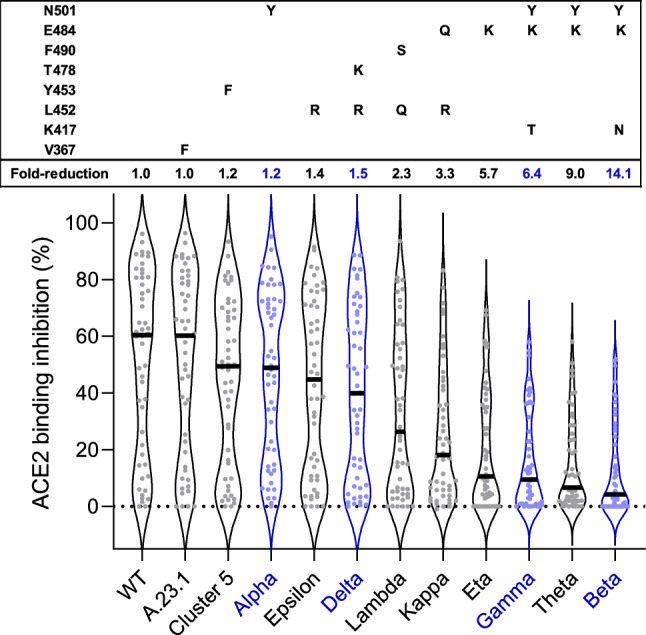
ACE2 binding inhibition varies between RBD mutants. Violin plots showing ACE2 binding inhibition (%) of individual serum samples from 7 to 49 days post PCR (n = 50, depicted as dots) against RBD mutants. Black horizontal lines represent medians. Fold-reduction of ACE2 binding inhibition in comparison to wild-type corresponds to the ratio between the medians of wild-type and the respective RBD mutant. VOC-RBDs are shown in blue. Mutations of each RBD mutant are shown in the box above the violin plot.

Having developed and validated RBDCoV-ACE2, as well as identifying superior performance to a commercially available kit, we then analyzed ACE2 binding inhibition within 266 serum samples from 168 COVID-19 patients (COVID-19 sample collection), including longitudinal samples from 35 donors. Samples were measured against RBD wild-type and 11 variants (hereafter referred to as “RBD mutants”) of SARS-CoV-2. All RBD mutants except A.23.1 showed reduced ACE2 binding inhibition compared to wild-type (1.2-fold (Cluster 5) to 14.1-fold (beta), Fig. 3) in serum samples taken within the first 49 days post initial positive PCR test. In the set of tested VOCs, alpha had the lowest reduction in ACE2 binding inhibition (1.2-fold), followed by delta (1.5-fold), gamma (6.4-fold) and beta (14.1-fold). While reduction in ACE2 binding inhibition was variant-specific, mutations at critical residues (e.g. E484K) appeared to have the largest effect (Fig. 3). Among the current and former VOIs, epsilon had the lowest reduction (1.4 fold), followed by lambda (2.3 fold), kappa (3.3 fold), eta (5.7 fold) and theta (9.0 fold).

### ACE2 binding inhibition correlates with antibody production against spike domains

**Figure 4:**
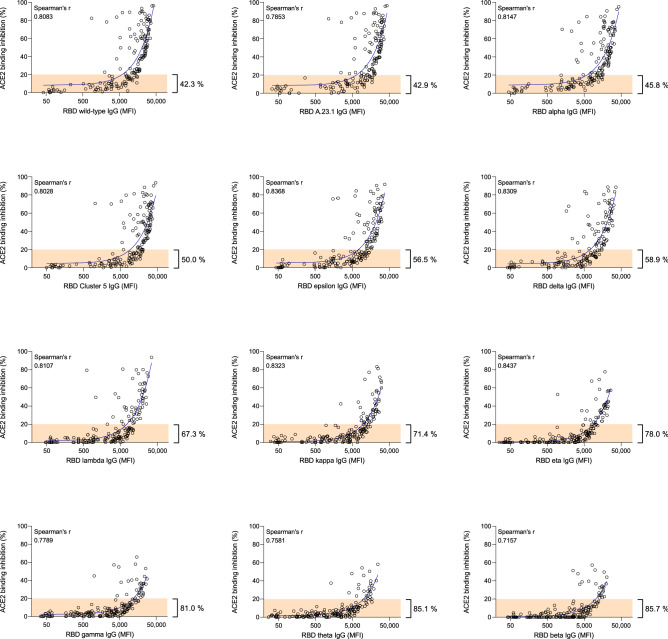
Correlation between anti-RBD IgG MFI signals and ACE2 binding inhibition (%) of serum samples from COVID-19 patients for wild-type and 11 RBD mutants. Regression analysis comparing ACE2 binding inhibition (%) and IgG responses (MFI) for wild-type and all RBD mutants included in the study. Each circle represents one sample (n = 168). For longitudinal donors with more than one sample available, the sample closest to 20 days post positive PCR diagnosis was selected. The percentage next to the bracket indicates the proportion of samples with ACE2 binding inhibition ≤ 20% (in orange). Spearman’s correlation coefficient (r) is specified for every correlation.

To determine if a correlation existed between ACE2 binding inhibition and RBD-specific antibody levels, we analyzed all samples with MULTICOV-AB^34^. ACE2 binding inhibition and SARS-CoV-2 RBD IgG antibody responses were positively correlated (all Spearman’s correlation coefficients above 0.70, Fig. 4) with variant-specific differences still present and reflecting. Additionally, we could show the positive correlation between ACE2 binding inhibition and S1/trimeric spike antibody production (Figure S3a and b). The ACE2 binding inhibitions of both S1 and trimeric spike coated beads compared to the inhibition of RBD beads were strongly correlated (all Spearman’s correlation coefficients above 0.95) (Figure S3c and d). In contrast, beads coated with the SARS-CoV-2 spike S2 domain, which does not interact with ACE2 in vivo, showed no ACE2 binding in our assay. Those findings confirm specific binding of ACE2 to its natural binding partners and therefore reaffirms that the presence of neutralizing antibodies is being detected. For all RBD mutants, the increase in ACE2 binding inhibition most commonly occurred once IgG RBD MFI levels exceeded 10,000 (Fig. 4). Notably, there was individual variation among the samples, with some having high ACE2 binding inhibition but relatively low IgG responses. For RBD mutants with a E484K mutation (eta, gamma, theta and beta), more than 78% of all samples were considered negative, compared to 42% for wild-type (Fig. 4).

### ACE2 binding inhibition decreases over time

**Figure 5:**
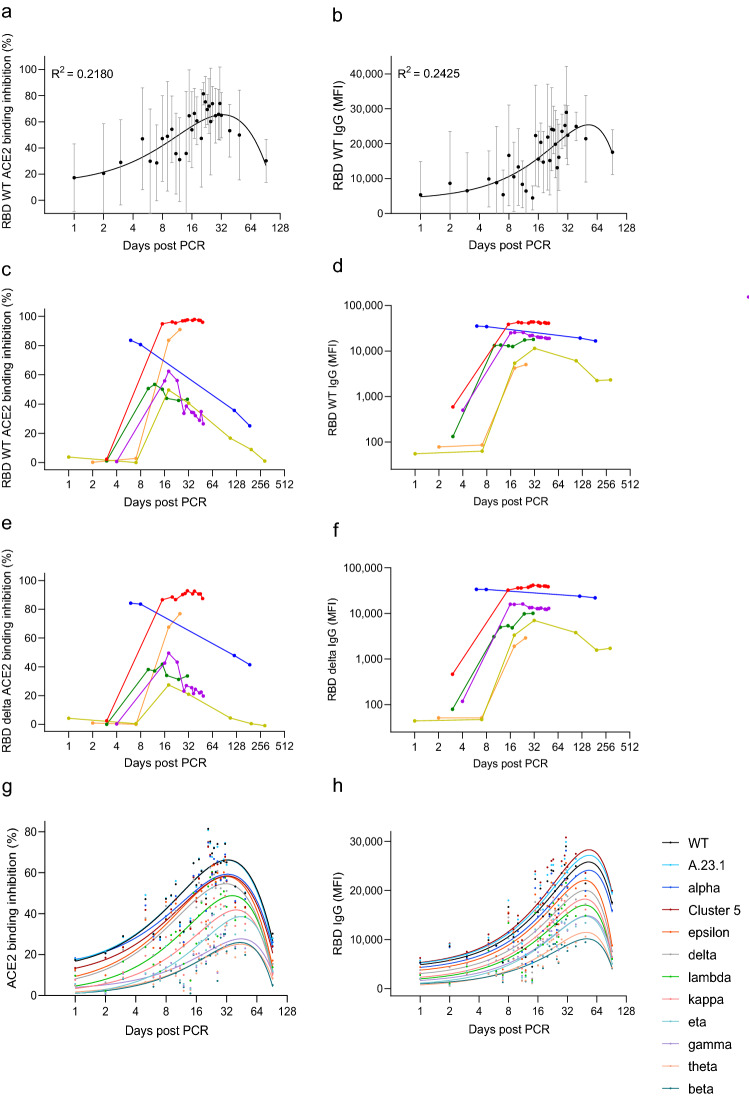
Longitudinal analysis of ACE2 binding inhibition and anti-RBD IgG levels in COVID-19 patients. Mean ACE2 binding inhibition (%) and IgG responses (MFI) for wild-type RBD against time post positive PCR test for samples (n = 149) taken from 1 to 92 days post PCR are shown (**a**,**b**). Black dots indicate mean responses with standard deviation indicated by the error bars. The same analysis is then shown for longitudinal samples of selected donors (n = 6) for wild-type (**c**,**d**) and RBD delta (**e**,**f**). For all RBD mutants, mean ACE2 binding inhibition (%) and mean IgG responses (MFI) 1 to 92 days post PCR are shown (**g**,**h**). Each variant is illustrated by a different color according to the figure key.

To examine whether ACE2 binding inhibition changes over time, we analyzed longitudinal samples from 35 study participants (range 1–290 days post-initial positive PCR). ACE2 binding inhibition and RBD antibody titers originally remained low directly following a positive test, before rapidly increasing (mean peak at day 23 post-PCR) and then decreasing (Fig. 5a,b). Due to the highly individualistic nature of the responses, we confirmed this pattern by analyzing a subset of six individuals with similar sample collection points (Fig. 5c,d). As delta represents the current dominant global strain, we then examined whether any differences in ACE2 binding inhibition and antibody binding were present within this variant compared to wild-type. Overall, ACE2 binding inhibition and IgG response followed the same pattern for all samples as for wild-type (Fig. 5e,f). We then confirmed that this pattern was true for all RBD variants (Fig. 5g,h). As expected, while there were differences in reduction in binding inhibition between the variants, all variants examined follow the same pattern of binding inhibition over time.

### ACE2 binding inhibition correlates with disease severity

**Figure 6:**
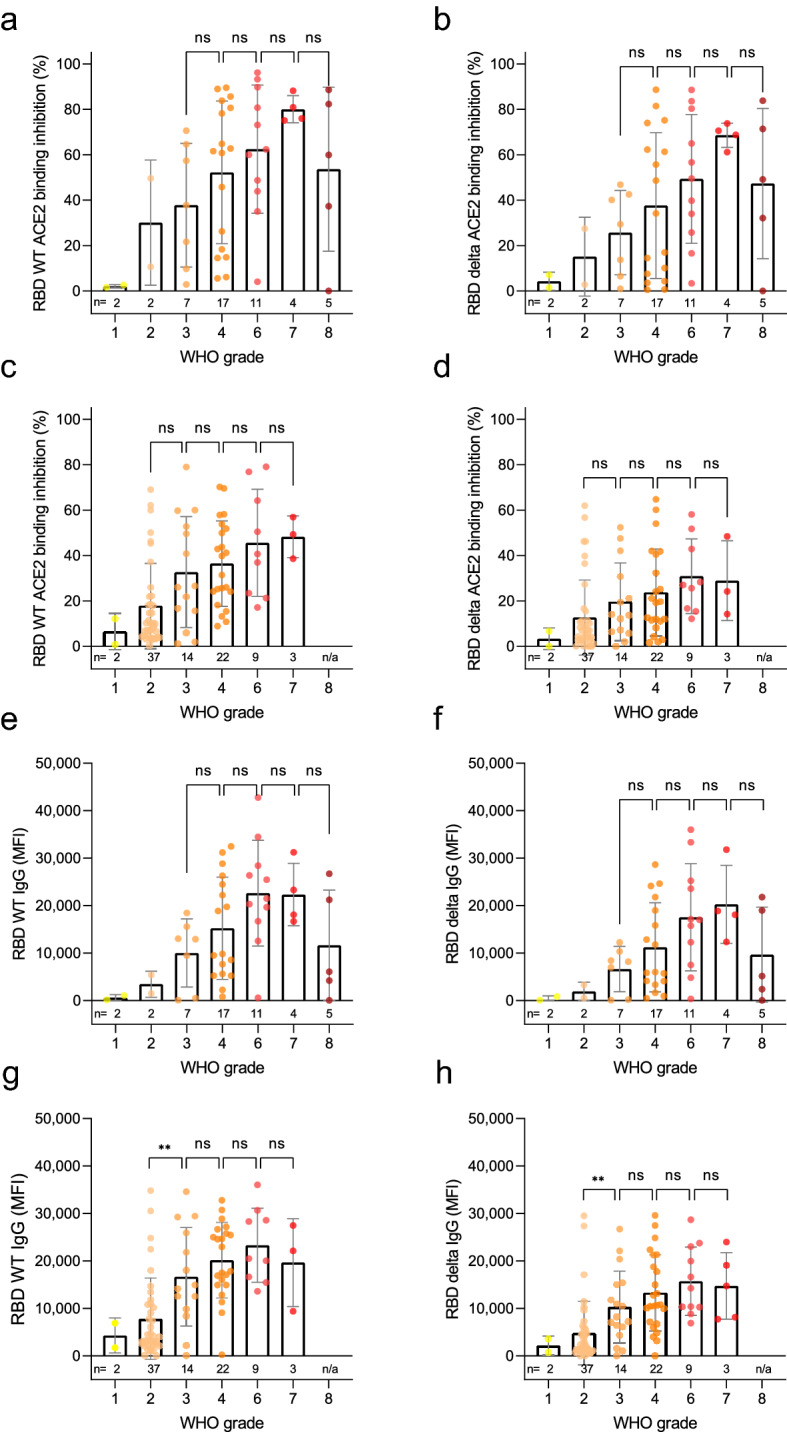
Correlation of anti-RBD IgG levels and ACE2 binding inhibition with SARS-CoV-2 disease severity. Bar charts showing mean ACE2 binding inhibitions (%) against wild-type and delta RBD are correlated with WHO grades for disease severity for samples 7–49 days post PCR (**a**,**b**) and ≥ 50 days post PCR (**c**,**d**). Mean anti-RBD WT IgG and anti-RBD delta IgG levels are shown for samples 7–49 days post PCR (**e**,**f**) and ≥ 50 days post PCR (**g**,**h**). Individual samples are displayed as colored dots, bars indicate the mean of the dataset with error bars representing standard deviation. Number of samples is given below the columns (n). If no samples for a group were available, the column is labeled with “n/a”. WHO grade 1—ambulatory/no limitations of activities, 2—ambulatory/limitation of activities, 3—hospitalized, mild disease/no oxygen therapy, 4—hospitalized, mild disease/mask or nasal prongs, 6—hospitalized, severe disease/intubation + mechanical ventilation, 7—hospitalized, severe disease/ventilation + additional organ support (pressors, RRT, ECMO), 8—Death. The study did not contain samples of WHO grade 5.

We then examined correlations between ACE2 binding inhibition and COVID-19 disease severity within our population of COVID-19 patients. The severity of COVID-19 infection was determined according to the WHO grading scale. For analysis purposes, samples were split into two separate timeframes, 7–49 days post-initial positive PCR and ≥ 50 days post-initial positive PCR, in order to examine both the log and lag stages of infection. While all WHO grades (except for 5 and 8 for samples ≥ 50 days post-initial positive PCR) were represented within both timeframes, the early log timeframe consisted mostly of samples in WHO grades 4 and 6, while the later lag timeframe samples were mostly WHO grades 2 to 4. ACE2 binding inhibition was examined for both WT and delta to confirm no differences between wild-type and the variants existed. Regardless of timeframe, ACE2 binding inhibition increased as disease severity increased. Within the early log timeframe, ACE2 binding inhibition for wild-type and delta RBD increased steadily with disease severity up to grade 7 (WHO grading scale, hospitalized patients needing intubation and mechanical ventilation), before decreasing for patients of grade 8 (fatal disease course) (Fig. 6a,b). Within the later lag timeframe, ACE2 binding inhibitions increased with disease severity (Fig. 6c,d), however there was an overall reduction for grades 4 to 7 compared to the early timeframe for both wild-type and delta (Fig. 6c,d). As expected, anti-RBD IgG levels also correlated with disease severity in both timeframes for wild-type and delta (Fig. 6e–h). Peak mean IgG levels were observed at grade 6 severity for wild-type and grade 7 for delta, 7–49 days post PCR. Post 49 days, mean IgG levels peaked for patients with grade 6 severity. As confirmation, confounding variables (age, gender, BMI) were examined for any potential effect on the results (Figure S4). While gender had no effect, we did find correlations between ACE2 binding inhibition and donor age for samples taken ≥ 50 days post-positive PCR (p = 0.0001), as well as BMI for samples collected in both timeframes (< 49 days p = 0.0330, ≥ 50 days p = 0.0017) (Figure S4d-f).

## Discussion

With vaccination campaigns now increasingly focusing on the role of booster doses, the quality of immune protection against SARS-CoV-2 in view of constantly emerging variants is of great interest. Whereas in the early-phase of the pandemic SARS-CoV-2 antibody assays were helpful in determining seroprevalence and support vaccine development, now a reliable correlate of immune protection is needed to securely lift social restrictions and guide future vaccine developments.

We show here that the performance of RBDCoV-ACE2 correlates strongly with classical VNTs, confirming that the assay is measuring the activity of neutralizing antibodies, while our technical validation also confirms RBDCoV-ACE2 is stable and reproducible. While cell-culture based VNTs (e.g. plaque reduction neutralization test) are the gold standard for neutralization assays, they have many disadvantages over conventional protein-based surrogate assays. Such assays require rapid access to continually changing virus variants and as such special biosafety level 3 laboratories are necessary. Additionally, VNTs are cell-culture based and therefore it takes multiple days to conduct an experiment with reproducibility potentially affected by either the cells or their long culture conditions. Consequently, highly reproducible assays under substantially faster and safer working conditions (e.g. BSL 1) would be highly beneficial. RBDCoV-ACE2 is finished in under 4 h and only requires 5 µL of patient sample to measure ACE2 binding inhibition simultaneously against multiple SARS-CoV-2 VOCs and VOIs. As a protein-based assay, it does not require enhanced safety protocols to be followed and can be completed safely in a BSL1 laboratory. Due to the bead-based nature and plate format, it is automatable, suitable for high-throughput, standardized and highly reproducible. The protein-based nature also allows for the rapid inclusion of emerging variants or single mutations. In comparison to the commercially available inhibition assay examined (NeutraLISA), RBDCoV-ACE2 did not have an apparent saturation phase, and therefore has a resolution range that enables greater separation of samples, particularly those that are strongly inhibiting ACE2 binding. The stronger correlation of RBDCoV-ACE2 to the VNT compared to NeutraLISA makes it a more accurate alternative to commercially available inhibition assays.

Similarly to other authors^35,36^, we identified a positive correlation between anti-RBD IgG levels and ACE2 binding inhibition, suggesting that neutralizing antibodies represent a consistent portion of all antibodies produced. Similar correlations between anti-S1 and anti-trimeric spike IgG levels and ACE2 binding inhibition as well as no ACE2 binding to the S2 domain reinforce this conclusion. There is, however, a large degree of individualism in responses, with some samples having low titers yet high ACE2 binding inhibition for specific RBD mutants. We also identified, as other have done previously^6,37^, a correlation between disease severity and ACE2 binding inhibition. However, the decrease in IgG levels and ACE2 binding inhibition of patients with WHO disease grade 8 (death) has not to our knowledge been reported before. This decrease requires further investigation to determine its cause, given its likely role in patient mortality.

As expected, ACE2 binding inhibition towards VOCs was highly variable. The strongest reductions in binding inhibition compared to wild-type were all from variants with a E484K mutation (eta, gamma, theta and beta). This specific mutation has been reported in multiple studies as an escape mutation that enhances the RBD-ACE2 affinity^38^. ACE2 binding inhibition was further reduced among these variants for those which additionally had a N501Y mutation (gamma, theta and beta), which is known to further enhance RBD-ACE2 binding^39^. These results are in-line with previous findings that have reported significant reductions in neutralization for gamma and beta^40–43^. The gamma and beta RBDs in our assays are only separated by a single K417N mutation, which is known to significantly reduce both the RBD-ACE2 binding affinity as well as the binding affinity to monoclonal therapeutic antibodies or other human antibodies^44^. Among recently emerged strains (delta, kappa, lambda), ACE2 binding inhibition compared to wild-type was reduced for all. The reduction in ACE2 binding inhibition seen for kappa and delta are comparable to recent findings^45^, although we could not confirm the reduction seen by other authors for Lambda^46^. This is likely due to the 7-amino acid deletion in the N-terminal domain of lambda’s spike protein, which is not present in the RBD and is thought to contribute to its immune evading properties^47^. Overall, the reduction in ACE2 binding inhibition against RBDs of all analyzed variants compared to wild-type has important implications for the design of second generation vaccines.

RBDCoV-ACE2 has limitations similar to other protein-based in vitro neutralization assays, such as only accounting for the Nabs that block the RBD-ACE2 interaction site through steric hindrance, and not for Nabs that interfere with cell entry mechanisms as would be analyzed in a VNT. Furthermore, the binding assay is also more prone to non-specific binding events. However, a major advantage of RBDCoV-ACE2 over VNTs is the speed of response toward viral evolution such as emerging variants of concern. The bead-based format of the assay is also highly reproducible and not susceptible to changes in experimental conditions, as is the case for cell culture based VNTs. The plate format of the assay also enables automation and high-throughput screening. Our assay only requires recombinant expressed RBD proteins, which can be quickly and easily produced. Additionally, this assay has the possibility of introducing artificial mutants to screen for possible escape variants that could arise in the future. Among our COVID-19 study population, the majority were admitted to the intensive care unit, meaning that the more serious grades of COVID-19 infection are heavily overrepresented in our population, while asymptomatic infections, which are known to be the most common form of disease progression^48^, are severely underrepresented. Our sample set for longitudinal analysis is also highly variable in sampling times post-PCR. However, this large variation is also beneficial as it clearly demonstrates the individual variability in ACE2 binding inhibition.

In conclusion, we have developed and validated RBDCoV-ACE2, an ACE2-RBD inhibition assay that analyzes current variants of concern/under investigation/interest of SARS-CoV-2. Assay performance showed good correlation to VNT, confirming that neutralizing antibodies are being analyzed. ACE2 binding inhibition was highly variable among all variants examined, with the 484 aa residue appearing to be critical in reducing ACE2 binding inhibition. ACE2 binding inhibition correlated with both antibody titers and disease severity, although responses were highly individualistic. Overall, the protein-based format of the assay allows for the fast and simple incorporation of new SARS-CoV-2 variants, enabling rapid screening to identify how ACE2 binding inhibition is altered for emerging variants, or in guiding next-generation vaccine development to target a range of SARS-CoV-2 variants.

## Materials and methods

### Sample collection for assay validation

16 serum samples consisting of 12 samples from COVID-19 patients (ethical approval #179/2020/BO2, University Hospital Tübingen) and four negative pre-pandemic samples (Central BioHub) were measured by both virus neutralization test and RBDCoV-ACE2 as part of the assay validation.

For technical assay validation, negative pre-pandemic serum samples were purchased from Central BioHub and four previously collected vaccinated samples from healthcare workers vaccinated with the Pfizer BNT-162b2 vaccine^30^ (222/2020/BO2, University Hospital Tübingen) as well as one sample from a COVID-19 patient (#179/2020/BO2, University Hospital Tübingen) were used.

### COVID-19 sample collection

266 serum samples were collected from 168 patients hospitalized at the University Hospital Tübingen, Germany, between April 17, 2020 and May 12, 2021. Longitudinal samples were measured from 35 of the 168 patients ranging from 2 to 12 samples per patient. All individuals were tested positive by SARS-CoV-2 PCR. Key characteristics of the study population are summarized in Table S1.

For serum collection, blood was extracted by venipuncture, with the serum blood collection tube rotated 180° two to three times to extract possible air bubbles in the sample. After a minimum coagulation time of 30 min at room temperature, serum was extracted by centrifugation for 15 min at 2000×*g* (RT) and then stored at − 80 °C until analysis. Time between blood sampling and centrifugation did not exceed 2 h.

### Ethical approval

Collection of samples and the execution of this study was approved by the Ethics committee of the Eberhard Karls University Tübingen and the University Hospital Tübingen under the ethical approval numbers 188/2020A and 764/2020/BO2 to Prof. Dr. Michael Bitzer. All participants signed the broad informed written consent of the Medical Faculty Tübingen for sample collection and all methods were performed in accordance with the relevant guidelines and regulations. Samples that were used for assay validation had their collection approved by the Ethics committee of the Eberhard Karls University Tübingen and the University Hospital Tübingen under the ethical approval numbers 222/2020/BO2 to Dr. Karina Althaus and 179/2020/BO2 to Prof. Dr. Juliane Walz. For collection of assay validation samples, informed written consent was obtained and all methods were performed in accordance with the relevant guidelines and regulations.

### Expression and purification of SARS-CoV-2 RBD mutants

The expression plasmid pCAGGS, encoding the receptor-binding domain (RBD) of SARS-CoV-2 spike protein (amino acids 319–541), was kindly provided by F. Krammer^49^. Expression and purification of VOCs alpha, beta and epsilon was carried out as previously described^30,50^. RBDs of SARS-CoV-2 VOCs gamma, delta, eta, theta, kappa and A.23.1 were generated by PCR amplification of fragments from wild-type or cognate DNA templates and subsequent fusion PCR by overlap extension to introduce described mutations. Based on RBD wild-type sequence, primer pairs RBDfor, E484Krev and E484Kfor, RBDrev for VOC eta and RBDfor, V367Frev and V367Ffor, RBDrev for A.23.1 were used. VOC lambda was generated based on RBD wild-type sequence using primer pairs L452Qfor, L452Qrev and F490Sfor, F490Srev. VOC delta was generated based on VOC epsilon using primer pairs RBDfor, T478Krev and T478Kfor, RBDrev. Based on VOC alpha sequence, VOC theta was generated using primer pairs RBDfor, E484Krev and E484Kfor, RBDrev. VOC kappa was generated based on VOC eta sequence using primer pairs RBDfor, L452Rrev and L452Rfor, RBDrev. VOC gamma was generated based on VOC theta sequence using primer pairs RBDfor, K417Trev and K417Tfor, RBDrev. Amplificates were inserted into the pCDNA3.4 expression vector using XbaI and NotI restriction sites. The integrity of all expression constructs was confirmed by standard sequencing analysis. An overview of the primer sequences is shown in Table S2. Confirmed constructs were expressed in Expi293 cells^30,34^. Briefly, cells were cultivated (37 °C, 125 rpm, 8% (v/v) CO_2_) to a density of 5.5 × 10^6^ cells/mL and diluted with Expi293F expression medium. Transfection of the corresponding plasmids (1 µg/mL) with Expifectamine was performed as per the manufacturer’s instructions. Enhancers were added as per the manufacturer’s instructions 20 h post transfection. Cell suspensions were cultivated for 2–5 days (37 °C, 125 rpm, 8% (v/v) CO_2_) and centrifuged (4 °C, 23,900 × *g*, 20 min) to clarify the supernatant. Afterwards, supernatants were filtered with a 0.22 µm membrane (Millipore, Darmstadt, Germany) and supplemented with His-A buffer stock solution (20 mM Na_2_HPO_4_, 300 mM NaCl, 20 mM imidazole, pH 7.4). The solution was applied to a HisTrap FF crude column on an Äkta pure system (GE Healthcare, Freiburg, Germany), extensively washed with His-A buffer, and eluted with an imidazole gradient (50–400 mM). Amicon 10 K centrifugal filter units (Millipore, Darmstadt, Germany) were used for buffer exchange to PBS and concentration of eluted proteins.

### Bead coupling

The in-house expressed RBD mutants were immobilized on magnetic MagPlex beads (Luminex) using the AMG Activation Kit for Multiplex Microspheres (# A-LMPAKMM-400, Anteo Technologies). In brief, 1 mL of spectrally distinct MagPlex beads (1.25 *10^7^ beads) were activated in 1 mL of AnteoBind Activation Reagent for 1 h at room temperature. The beads were washed twice with 1 mL of conjugation buffer using a magnetic separator, before being resuspended in 1 mL of antigen solution diluted to 25 µg/mL in conjugation buffer. After 1 h incubation at room temperature the beads were washed twice with 1 mL conjugation buffer and incubated for 1 h in 0.1% (w/v) BSA in conjugation buffer for blocking. Following this, the beads were washed twice with 1 mL storage buffer. Finally, the beads were resuspended in 1 mL storage buffer and stored at 4 °C until further use.

### RBDCoV-ACE2

Assay buffer (1:4 Low Cross Buffer (Candor Bioscience GmbH) in CBS (1 × PBS + 1% BSA) + 0.05% Tween20) was supplemented with biotinylated human ACE2 (Sino Biological, # 10108-H08H-B) to a final concentration of 342.9 ng/mL to produce ACE2 buffer. Working inside a sterile laminar flow cabinet, serum samples were thawed and diluted 1:25 in assay buffer before being further diluted 1:8 in ACE2 buffer resulting in a final concentration of 300 ng/mL ACE2 in all 1:200 diluted samples. Spectrally distinct populations of MagPlex beads (Luminex) coupled with RBD proteins of SARS-CoV-2 wild-type and variants alpha, beta, gamma, epsilon, eta, theta, kappa, delta, lambda, Cluster 5 and A.23.1 were pooled in assay buffer to create a bead mix (40 beads/µL per bead population). 25 µL of diluted serum was added to 25 µL of bead mix in each well of a 96-well plate (Corning, #3642). To allow comparison of ACE2 binding inhibition between different RBD mutants on a relative scale, 300 ng/mL ACE2 without added serum was measured in triplicates on every plate as normalization control. Additionally, one quality control sample was analyzed in triplicates on every plate. For blank measurement, 25 µL assay buffer instead of diluted sample was added to two wells per plate. Samples were incubated for 2 h at 21 °C while shaking at 750 rpm on a thermomixer. Following incubation, the beads were washed three times with 100 µL wash buffer (1 × PBS + 0.05 Tween20) using a microplate washer (Biotek 405TS, Biotek Instruments GmbH). For detection of bound biotinylated ACE2, 30 µL of 2 µg/mL RPE-Streptavidin was added to each well and the plate was incubated for 45 min at 21 °C while shaking at 750 rpm on a thermomixer. Afterwards, the beads were washed again three times with 100 µL wash buffer. The 96-well plate was placed for 3 min on the thermomixer at 1000 rpm to resuspend the beads before analysis using a FLEXMAP 3D instrument (Luminex) with the following settings: 80 µL (no timeout), 50 events, Gate: 7500–15,000, Reporter Gain: Standard PMT. MFI values of each sample were divided by the mean of the ACE2 normalization control. The normalized values were converted into percent and subtracted from 100 resulting in the percentage of ACE2 binding inhibition. Negative values were manually set to zero.

### MULTICOV-AB

MULTICOV-AB^34^, an in-house produced SARS-CoV-2 antibody assay, was performed with all serum samples to measure RBD/S1/trimeric spike-specific IgG levels. The antigen panel was expanded to include RBD proteins from 11 different SARS-CoV-2 variants from which all, except the Cluster 5 variant from Sino Biological (# 40592-V08H80), were produced in-house. The assay was carried out as previously described^30^.

### Viruses

All experiments associated with the SARS-CoV-2 virus were conducted in a Biosafety Level 3 laboratory. The recombinant infectious SARS-CoV-2 clone expressing mNeonGreen (icSARS-CoV-2-mNG)^51^, corresponding to the 2019-nCoV/USA_WA1/2020 isolate, was obtained from the World Reference Center for Emerging Viruses and Arboviruses (WRCEVA) at the UTMB (University of Texas Medical Branch). The mNeon Green reporter gene introduced into ORF7 allows the differentiation between infected and uninfected cells.

The generation of icSARSCoV-2-mNG stocks and the MOI determination was performed as previously described^52^.

### Virus Neutralization Assay (VNT)

VNTs were determined previously^53^. Briefly, 1 × 10^4^ Caco-2 cells/well were seeded in 96-well plates the day before infection in media containing 5% FCS. Caco-2 cells were co-incubated with the SARS-CoV-2 strain icSARS-CoV-2-mNG^51^ at a MOI = 1.1 and serum samples in two-fold serial dilutions ranging from 1:40 to 1:5120. 48 h post infection, cells were fixed with 2% PFA and stained with Hoechst33342 (1 µg/mL final concentration) for 10 min at 37 °C. Following this, the staining solution was removed and exchanged for PBS. To quantify infection rates, images were taken with the Cytation3 (Biotek Instruments GmbH) and Hoechst + and mNG + cells were automatically counted by the Gen5 Software (Biotek Instruments GmbH). Infection rate was determined by dividing the number of infected cells through total cell count per condition. Virus-neutralizing titers (VNT50s) were calculated as the half-maximal inhibitory serum dilution.

### Assay validation experiments

To determine the intra-assay precision of RBDCoV-ACE2, 12 replicates of four serum samples (Vac1–Vac4) were measured on a 96-well plate (Corning, #3642). Additionally, 15 replicates of the 300 ng/mL ACE2 normalization control and 12 replicates of the blank control containing only assay buffer without sample or ACE2 were measured. For inter-assay precision, five serum samples (Vac1–Vac4 and Inf1) were measured in triplicates in four independent experiments. Additionally, the quality control, the ACE2 normalization control and blank were also processed in triplicates in the same four experiments. Short-term stability was determined by storing ACE2 buffer under six different conditions before proceeding with the assay protocol. The prepared ACE2 buffer was stored 2 h, 4 h and 24 h at both 4 °C and room temperature and compared to ACE2 buffer without storage (fresh). Replicate MFI values of every sample (Vac1–Vac4 (vaccinated), Inf1 (infected) and pre-pandemic) were normalized to the values of the respective ACE2 normalization control. Freeze–thaw stability of the biotinylated ACE2 stocks was determined by analyzing six serum samples (Vac1–Vac4 (vaccinated), Inf1 (infected) and pre-pandemic) in triplicates, with ACE2 stocks undergoing 1 to 5 cycles. In addition to that, every sample was also processed with ACE2 not re-frozen once thawed (fresh, 0 freeze–thaw cycles). The MFI values of every sample were normalized to the values of the respective ACE2 normalization control. To investigate the stability of RBDCoV-ACE2 against variations of the used ACE2 concentration, six samples (Vac1–Vac4 (vaccinated), Inf1 (infected) and pre-pandemic) were analyzed with ACE2 concentrations ranging from 150 ng/mL to 350 ng/mL. Replicate MFI values of every sample were normalized to the values of the respective ACE2 normalization control. For analysis, the mean, standard deviation and coefficient of variation in percent of all replicates were calculated.

To confirm that the multiplex assay format has no undesirable effect on ACE2 binding inhibition values compared to singleplex measurements, 24 samples (pre-pandemic (n = 5) and COVID-19 infected (n = 19)) were analyzed in both singleplex and multiplex (for all VOCs).

### NeutraLISA

One sample from each individual donor (n = 168) was analyzed with the commercially available in-vitro diagnostic test SARS-CoV-2 NeutraLISA (Euroimmun). The assay was performed according to the manufacturer’s instructions. For longitudinal donors with more than one sample available, the sample closest to 20 days after positive PCR diagnosis was picked. Negative values were manually set to zero.

### Statistical analysis

Data collection and assignment to metadata was performed with Microsoft Excel 2016. Data analysis, visualization and curve fitting was performed with Graphpad Prism (version 9.1.2). Virus-neutralizing titers (VNT50s) as the half-maximal inhibitory serum dilution were calculated using 4-parameter nonlinear regression. Longitudinal curves were fitted using a one-site total binding equation. Correlations were analyzed using Spearman’s correlation coefficient. Significances were calculated (where appropriate) using Mann–Whitney U tests. Figures were edited with Inkscape (version 0.92.4). Data generated for this manuscript is available from the authors upon request.

## Supplementary Information

Supplementary Information.
