# Supplementary material for: COVID-19 patient serum less potently inhibits ACE2-RBD binding for various SARS-CoV-2 RBD mutants
Source: Sci Rep. 2022 May 3;12:7168. doi: 10.1038/s41598-022-10987-2 (PMC9062870; doi:10.1038/s41598-022-10987-2)
Supplement: Supplementary file 1 — Supplementary Information. [file 41598_2022_10987_MOESM1_ESM.pdf]

COVID-19 patient serum less potently inhibits ACE2-RBD binding for various SARS-CoV-2 RBD mutants

Daniel Junker[1], Alex Dulovic[1], Matthias Becker[1], Teresa R. Wagner[1,2], Philipp D. Kaiser[1], Bjoern Traenkle[1], Katharina Kienzle[3], Stefanie Bunk[3], Carlotta Struemper[3], Helene Haeberle[4], Kristina Schmauder[5,6], Natalia Ruetalo[7], Nisar Malek[3,8], Karina Althaus[9], Michael Koeppen[4], Ulrich Rothbauer[1,2], Juliane S. Walz[10,11,12,13], Michael Schindler[7], Michael Bitzer[3,8], Siri Göpel[3,6]*, Nicole Schneiderhan-Marra[1]*

Author Affiliations

[1] NMI Natural and Medical Sciences Institute at the University of Tübingen, Reutlingen, Germany

[2] Pharmaceutical Biotechnology, Eberhard Karls University, Tübingen, Germany

[3] Department Internal Medicine I, University Hospital Tübingen, Tübingen, Germany

[4] Department of Anesthesiology and Intensive Care Medicine, University Hospital Tübingen, Tübingen, Germany

[5] Institute for Medical Microbiology and Hygiene, University Hospital Tübingen, Tübingen, Germany

[6] German Centre for Infection Research (DZIF), Partner Site Tübingen, Tübingen, Germany

[7] Institute for Medical Virology and Epidemiology, University Hospital Tübingen, Tübingen, Germany

[8] Center for Personalized Medicine, Eberhard Karls University, Tübingen, Germany

[9] Institute for Clinical and Experimental Transfusion Medicine, University Hospital Tübingen, Tübingen, Germany

[10] Clinical Collaboration Unit Translational Immunology, German Cancer Consortium (DKTK), Department of Internal Medicine, University Hospital Tübingen, Tübingen, Germany

[11] Institute for Cell Biology, Department of Immunology, University of Tübingen, Tübingen, Germany

[12] Cluster of Excellence iFIT (EXC2180) "Image-Guided and Functionally Instructed Tumor Therapies", University of Tübingen, Tübingen, Germany

[13] Dr. Margarete Fischer-Bosch-Institute for Clinical Pharmacology and Robert Bosch Center for Tumor Diseases (RBCT), Stuttgart, Germany

Running Head: Less potent ACE2 binding inhibition against SARS-CoV-2 RBD mutants

* denotes shared senior authorship and corresponding authors.

Contact Information

Nicole Schneiderhan-Marra – Phone number +49 (0)7121 51530 815. Email Address Nicole.schneiderhan@nmi.de Postal Address – Markwiesenstrasse 55, 72770 Reutlingen, Germany.

Siri Göpel – Phone number +49 (0)7071 29 85415. Email Address siri.goepel@med.uni-tuebingen.de Postal Address – Otfried-Müller-Strasse 10, 72076 Tübingen, Germany.

Table S1 - Characteristics of the analyzed COVID-19 serum sample collection set.

**Characteristic**	
Number of donors	168
Number of samples	266
Median age (IQR) -years	62 (23)
Female sex (%)	78 (46.4)
Median ΔT post first positive PCR test (range)	91 (1-348)
Median BMI (range)	26.9 (18.4 – 50.2)

Table S2 - Primer sequences used for expression of RBD mutants.

Primer Name	Sequence (5‘ to 3‘)
RBDfor	ATATCTAGAGCCACCATGTTCGTGTTTCTGG
E484Krev	GCAGTTGAAGCCTTTCACGCCGTTACAAGGGGT
E484Kfor	GTAACGGCGTGAAAGGCTTCAACTGCTACTTCCC
RBD rev	AAGATCTGCTAGCTCGAGTCGC
V367Frev	CGGAGTTGTACAGGAAGGAGTAGTCGGCCACGCA
V367Ffor	CGACTACTCCTTCCTGTACAACTCCGCCAGCTTC
L452Qfor	GGCAACTACAATTACCAGTACCGGCTGTTCCGGAAG
L452Qrev	CGGTACTGGTAATTGTAGTTGCCGCCG
F490Sfor	TCAACTGCTACTCCCCACTGCAGTCCTACGGC
F490Srev	CTGCAGTGGGGAGTAGCAGTTGAAGCCTTCCAC
T478Krev	CGTTACAAGGCTTGCTGCCGGCCTGATAGA
T478Kfor	CCGGCAGCAAGCCTTGTAACGGCGTGGAAG
L452Rrev	CGGTACCGGTAATTGTAGTTGCCGCCG
L452Rfor	GGCAACTACAATTACCGGTACCGGCTGTTCCGGAAG
K417Trev	GTTGTAGTCGGCGATGGTGCCTGTCTGTCCAGGGG)
K417Tfor	GACAGACAGGCACCATCGCCGACTACAACTACAAG

Table S3 - RBDCoV-ACE2 technical validation results. Percentage coefficients of variation (%CV) of normalized MFI values for every SARS-CoV-2 RBD for all analyzed samples (n=6) including the mean of all samples.

	Samples	RBD WT	alpha	beta	gamma	epsilon	Cluster 5	A.23.1	eta	theta	kappa
**Intra-assay precision (%CV)**	Vac1	1.4	1.5	2.6	2.5	1.3	1.5	1.7	1.4	2.1	1.1
	Vac2	2.2	2.1	1.1	1.6	1.7	1.2	1.5	1.9	1.6	2.0
	Vac3	2.8	4.0	3.8	2.1	4.2	3.0	3.5	2.1	1.6	4.3
	Vac4	2.2	2.1	3.4	1.9	2.0	2.0	2.3	1.7	2.1	2.4
	**Mean**	**2.2**	**2.4**	**2.7**	**2.0**	**2.3**	**1.9**	**2.3**	**1.8**	**1.9**	**2.5**
**Inter-assay precision (%CV)**	Vac1	1.9	2.7	3.0	2.9	2.8	2.3	3.5	2.7	2.9	2.9
	Vac2	2.6	3.0	3.9	2.8	3.5	2.4	2.7	3.0	2.1	3.0
	Vac3	5.7	6.9	6.9	3.5	6.4	4.6	5.5	4.9	3.6	5.2
	Vac4	4.4	3.5	4.6	2.9	4.3	2.5	1.9	3.0	2.7	3.3
	Inf1	1.9	2.5	2.9	2.9	2.8	2.4	1.6	1.8	1.8	2.4
	**Mean**	**3.3**	**3.7**	**4.3**	**3.0**	**4.0**	**2.8**	**3.0**	**3.1**	**2.6**	**3.4**
**Short-term stability (%CV)**	Vac1	2.5	3.9	3.2	2.3	2.5	2.3	2.6	2.1	2.8	3.2
	Vac2	2.3	3.1	4.5	2.3	2.3	2.8	2.6	2.0	2.4	2.6
	Vac3	8.6	9.3	6.4	3.0	6.9	4.4	4.9	7.1	3.2	7.3
	Vac4	11.6	4.9	3.4	3.2	3.0	2.4	3.1	3.2	2.3	3.3
	Inf1	1.4	2.6	3.5	2.5	1.9	2.4	2.2	1.8	1.7	1.7
	Pre-pandemic	1.6	2.3	3.7	1.9	1.9	1.8	2.2	1.8	2.4	2.1
	**Mean**	**4.7**	**4.4**	**4.1**	**2.5**	**3.1**	**2.7**	**2.9**	**3**	**2.5**	**3.4**
**Freeze-thaw stability (%CV)**	Vac1	2.7	3.8	4.5	3.1	4.0	2.7	3.3	2.1	2.9	3.2
	Vac2	4.0	3.2	2.5	2.5	3.8	2.8	2.9	2.6	3.2	2.9
	Vac3	9.3	10.2	4.7	5.8	12.1	8.9	7.3	9.8	5.5	10.6
	Vac4	7.6	6.2	6.1	5.3	7.2	5.8	6.6	6.3	4.9	6.3
	Inf1	3.6	2.6	4.1	3.1	3.1	2.9	2.4	2.5	2.4	3.5
	Pre-pandemic	1.8	1.8	3.2	3.7	1.8	1.4	2.1	2.3	2.3	2.0
	**Mean**	**4.8**	**4.6**	**4.2**	**3.9**	**5.3**	**4.1**	**4.1**	**4.3**	**3.5**	**4.8**
**Parallelism (%CV)**	Vac1	4.1	3.9	5.0	4.0	4.5	4.2	4.2	3.2	2.6	3.9
	Vac2	4.2	2.5	4.0	3.4	2.7	2.5	4.2	2.6	2.8	4.2
	Vac3	12.0	11.7	4.0	5.3	11.9	11.8	9.3	13.6	4.5	12.7
	Vac4	11.3	9.6	6.5	6.9	10.0	8.7	8.6	9.9	4.4	9.3
	Inf1	5.2	3.4	5.5	3.4	4.8	4.1	4.0	4.2	4.2	3.6
	Pre-pandemic	2.1	1.2	3.6	2.8	2.3	2.1	2.4	1.9	2.7	1.4
	**Mean**	**6.5**	**5.4**	**4.8**	**4.3**	**6.0**	**5.6**	**5.5**	**5.9**	**3.5**	**5.9**

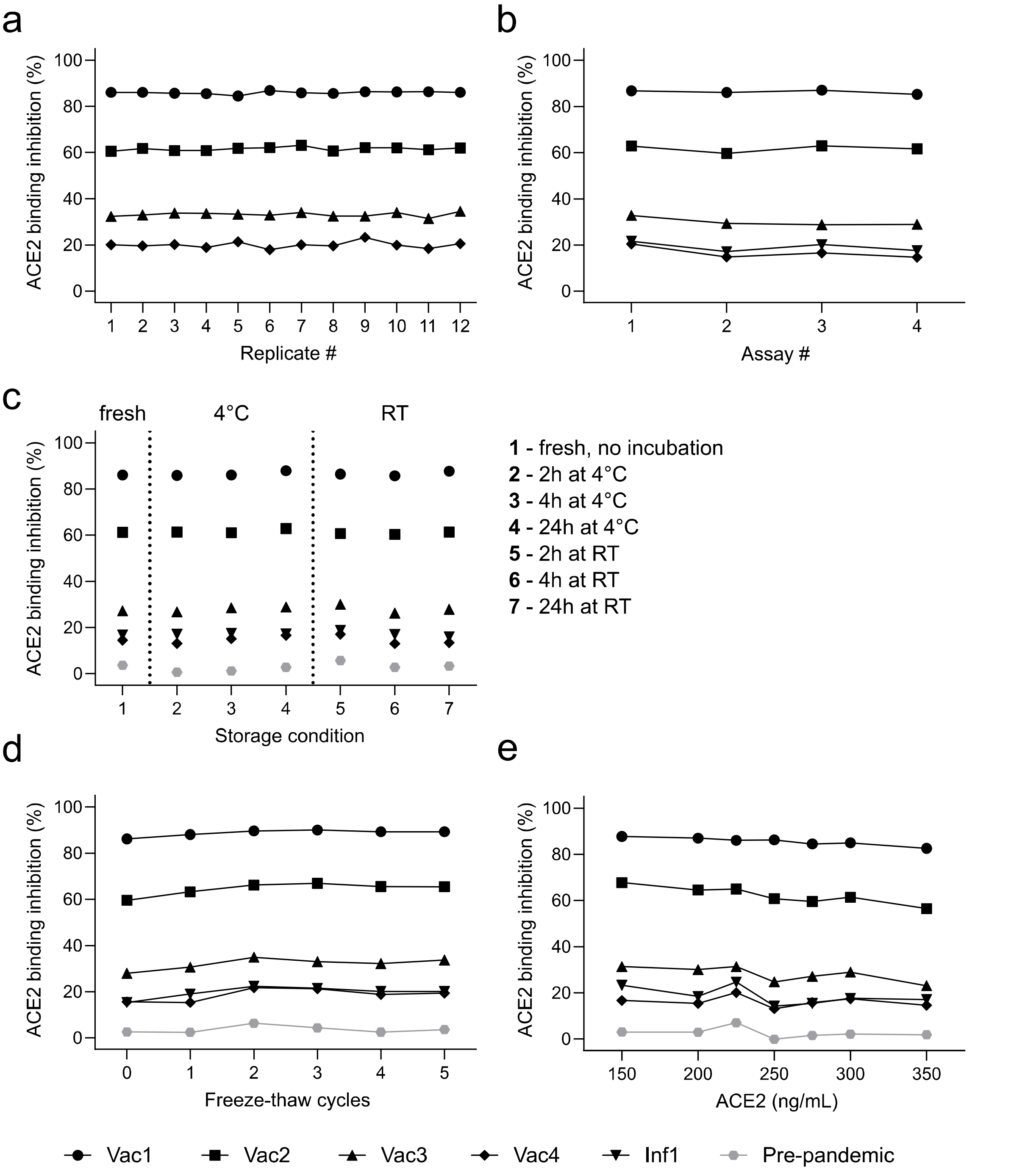

Figure S1 - RBDCoV-ACE2 technical validation results. Results of intra-assay precision (a), inter-assay precision (b), short-term stability (c), freeze-thaw stability (d) and parallelism (e) experiments analyzing ACE2 binding inhibition (displayed as %) using wild-type (WT) RBD. Four samples from donors vaccinated with Pfizer BNT-162b2 (n=4), one COVID-19 infected (n=1) and one pre-pandemic sample (n=1, grey) were analyzed. Data points of each sample are illustrated by different shapes according to the figure key. Percent coefficients of variation (%CV) for all included RBD mutants are summarized in **Table S3**.

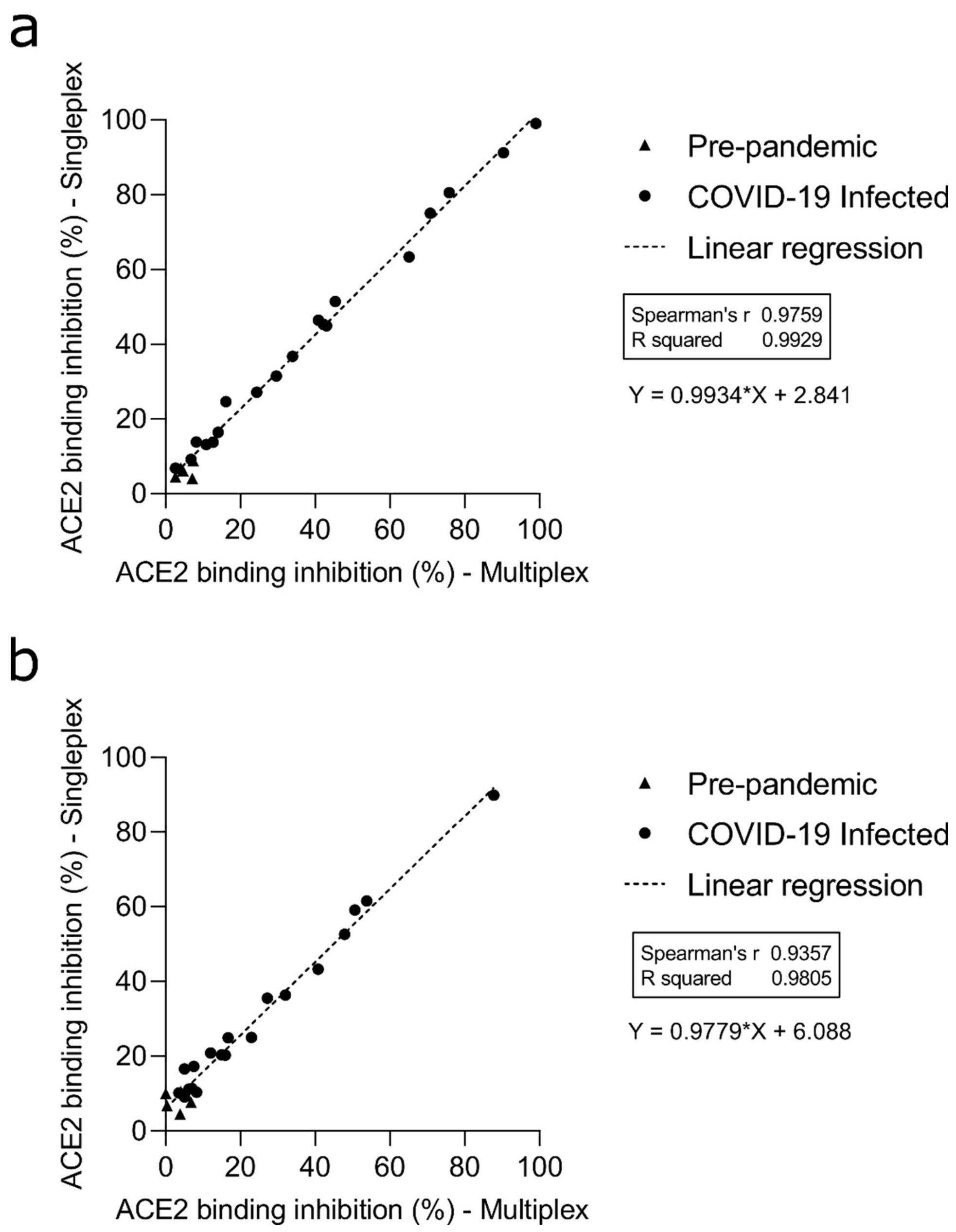

Figure S2 - Comparison of multiplex and singleplex assay formats. Linear regression analysis between ACE2 binding inhibition (%) values of samples from pre-pandemic (n=5) and COVID-19 infected (n=19) individuals analyzed in both multiplex and singleplex for RBD WT (a) and RBD delta (b). Correlation analysis was performed after Spearman and the correlation coefficient r is shown.

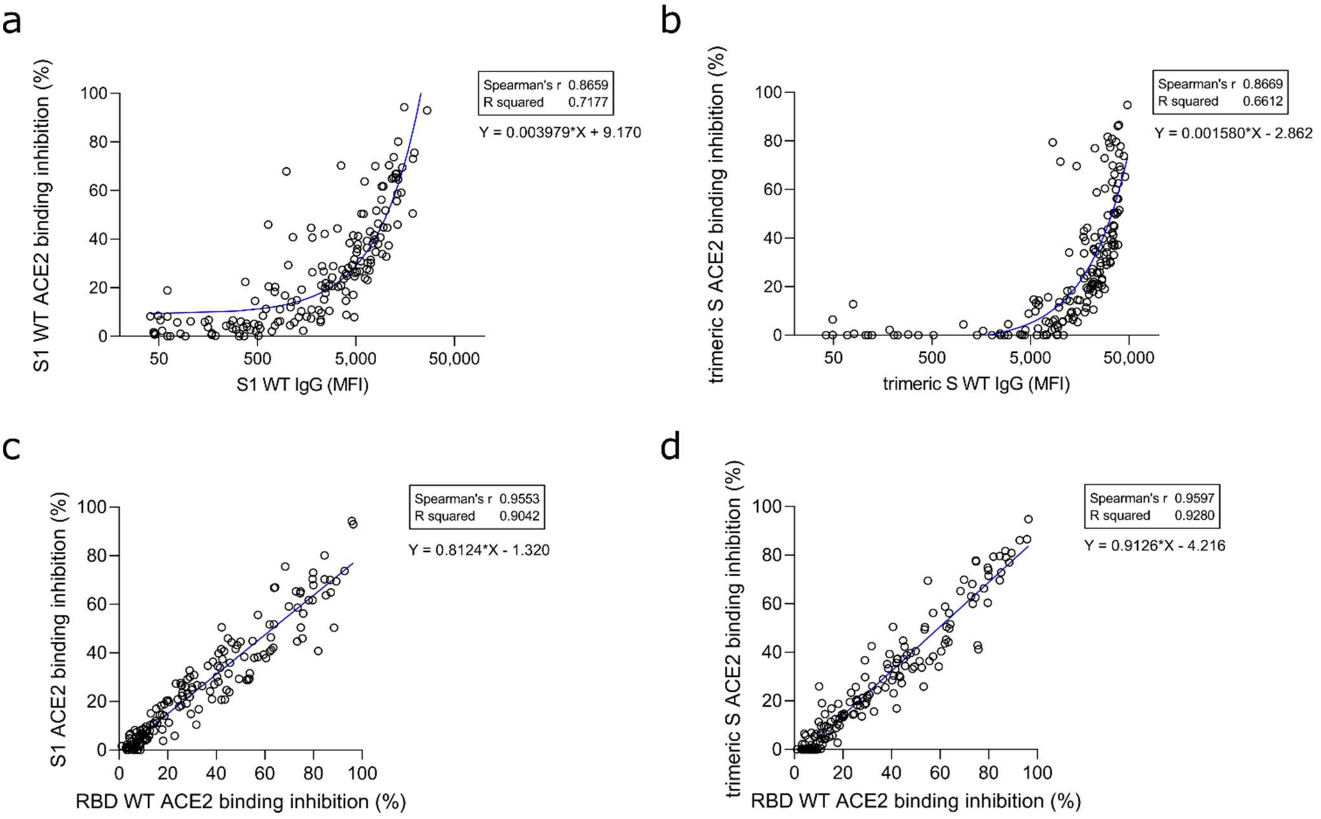

Figure S3 - Correlation between IgG MFI signals and ACE2 binding inhibition (%) against SARS-CoV-2 S1-domain (a) and trimeric spike (b) of serum samples from COVID-19 patients (n= 168). Regression analysis comparing ACE2 binding inhibitions (%) for S1 and trimeric spike with RBDCoV-ACE2 results of RBD WT (c and d). Each circle represents one sample (n=168). For longitudinal donors with more than one sample available, the sample closest to 20 days post positive PCR diagnosis was selected. Spearman’s correlation coefficient (r) as well as the equation of the linear regression is specified for every correlation.

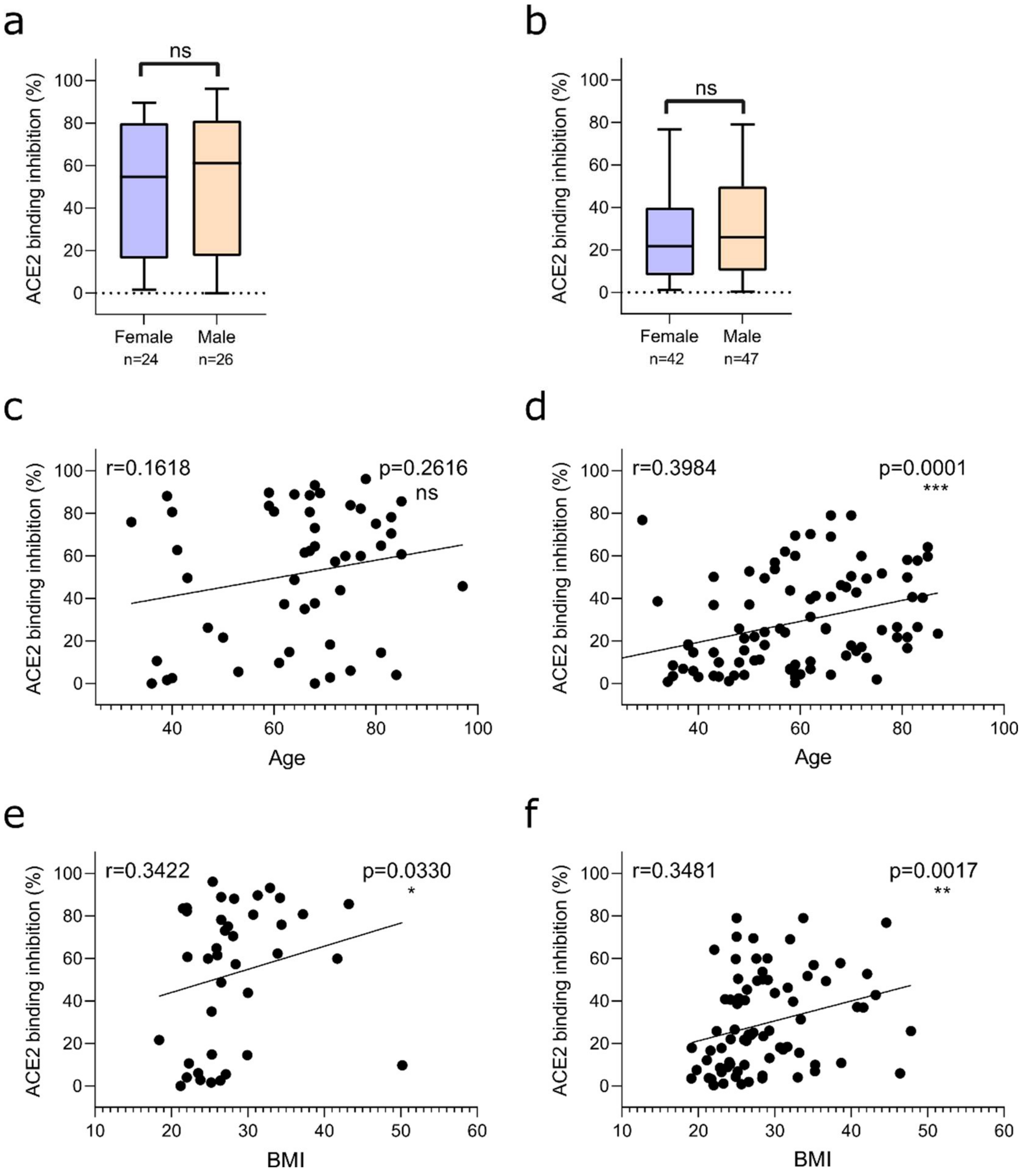

Figure S4 - Relation between ACE2 binding inhibition (%) and gender, donor age and Body-mass-index (BMI). Correlation between wild-type ACE2 binding inhibition (%) and gender (a, b), age (c, d) and BMI (e, f) for samples 7-49 days post PCR (a, c, e) and ≥ 50 days post PCR (b, d, f). P-values, when significant, are shown for all panels. Spearman's r was used to determine correlations.
